# Circulating neurofilaments to track dorsal root ganglion toxicity risks with AAV-mediated gene therapy

**DOI:** 10.1016/j.omtm.2022.06.005

**Published:** 2022-06-20

**Authors:** Michelle A. Farrar, Ewout Groen, Christiano R.R. Alves

**Affiliations:** 1 Department of Neurology, Sydney Children’s Hospital Network, Level 8, Bright Alliance Building, Avoca Street, Randwick, NSW 2031, Australia; 2 Discipline of Paediatrics and Child Health, School of Clinical Medicine, UNSW Medicine and Health, UNSW Sydney, Kensington, NSW 2052, Australia; 3 Department of Neurology, UMC Utrecht Brain Center, 3584 CX Utrecht, the Netherlands; 4 Department of Neurology, Massachusetts General Hospital, Harvard Medical School, Boston, MA 02114, USA; 5 Center for Genomic Medicine, Simches Research Center, Massachusetts General Hospital, Boston, MA 02114, USA

**Keywords:** adeno-associated virus, dorsal root ganglion, gene therapy, neurofilament, toxicity

## Abstract

Identifying non-invasive biomarkers is critical to evaluate the long-term safety of adeno-associated virus (AAV)-mediated therapies. Plasma neurofilament light chain (Nf-L) levels are associated with dorsal root ganglia toxicity in rats and monkeys, suggesting that circulating Nf-L is a promising tool to be included in clinical trials and practice.

Alongside the recent remarkable clinical reality of AAV gene therapy for serious diseases such as spinal muscular atrophy, preclinical and clinical studies have demonstrated important safety concerns, including dorsal root ganglion (DRG) toxicity. Toxicity risks of AAV gene therapy necessitate identification and inclusion of appropriate assessment strategies across the gene-therapy pipeline, amid continuing growth in potential applications within preclinical and clinical research. In this issue of *Molecular Therapy* - *Methods & Clinical Development*, Fader et al. identify plasma Nf-L as a potential non-invasive biomarker to monitor the development of DRG toxicity after AAV-mediated gene therapy.^1^

Fader et al.’s characterization of DRG toxicity after AAV gene therapy is concordant with recent reports in rodent and non-human primate models,^2^ providing a solid foundation for biomarker development. Adverse findings were impacted by dose, age, and route of administration. The AAV-mediated pathologies were evident in cervical, thoracic, and lumbar DRG, characterized by minimal to moderate infiltration of mononuclear inflammatory cells, proliferating resident satellite cells, and sensory neuronal degeneration. In addition, minimal to moderate axonal degeneration was identified in peripheral nerves and ascending dorsal tracts. Pathologies were mostly mild to moderate in severity and not associated with clinical signs of neuropathic pain or ataxia. Notably, another study reported proprioceptive deficits and ataxia in several piglets 14 days after administration of AAV containing the *SMN* transgene.^3^ DRG findings following AAV gene therapy were also demonstrated to be time dependent.

Fader et al. used a targeted approach to select Nf-L and ubiquitin C-terminal hydrolase (UCH-L1) as candidate biomarkers, based on commercial reagent availability, antibody cross reactivity, assay performance, and DRG protein expression.^1^ Dose- and time-dependent changes were evaluated after AAV gene therapy, comparing protein profiles with DRG pathology. Serum/plasma Nf-L levels were strongly associated with the severity of neuronal degeneration and axonal loss, with elevations commencing from day 8 in rodents and day 14 in monkeys. In comparison, cerebrospinal fluid (CSF) Nf-L levels demonstrated a weak association with DRG pathology. UCH-L1 levels were below quantification levels in most studies, and when detected, there was no or minimal DRG pathology. Based on these results, the practical challenges in CSF sampling (procedure-related neurological injury and obtaining sufficient volumes), and the location of DRG outside the blood-brain barrier, circulating NfL is most promising as a biomarker of DRG toxicity following AAV gene therapy. Pertinently, DRG pathology extended to glial and inflammatory cells, suggesting a broader repertoire of biomarkers, merits further consideration.

Biomarkers may function to further elucidate the causality and underlying cellular mechanisms of AAV-mediated DRG toxicity and the complex interplay between factors and investigate risk mitigation strategies. While Fader et al. demonstrate dose-dependent toxicity up to 1 × 10^14^ vg/kg, the *SMN* transgene cassette was constant, and several clinical trials are administering higher doses. Significantly, a study involving SMNΔ7 mice indicated that long-term overexpression of the SMN transgene caused cellular stress and neurodegeneration.^4^ A further study reported prevention of neuronal degeneration and axonopathy when using AAV gene therapy designed to include a specific microRNA target sequence that reduced transgene expression in DRG.^5^ Moreover, a recent study comparing different AAV9 production methods contained an AAV9-only control that did not cause DRG toxicity, indicating that toxicity is mediated specifically by transgene overexpression.^6^ Importantly, it may be challenging to directly compare the outcomes of studies in which similar, but slightly different, AAV types are used, as each AAV vector has its own characteristics and cell-type and tissue specificity.

As cytoplasmic proteins are abundantly expressed in axons, Nfs are candidate biomarkers in a range of neurological disorders associated with axonal degeneration. Consequently, Nf-L levels may be independently disrupted by underlying neurological conditions, complicating interpretation. Observations in preclinical studies suggest that DRG pathology may be a direct consequence of SMN depletion, independent of gene therapy.^7^ Pathological processes that occur concurrently with downstream consequences of gene therapy could further complicate the analysis of biomarker studies. Future assessments of the performance of plasma Nf-L to indicate neuroaxonal injury and severity with AAV vectors should consider comparisons with different transgenes and delineation from primary pathogenic processes.

The clinical significance and relative sensitivity of AAV DRG toxicity for humans remains unknown. Thus far, DRG toxicity has been reported in 3 participants enrolled in 2 clinical trials.^8^^,^^9^ An immune response in the DRG was linked to 2 adults who received intrathecal AAVrh10miRNA-SOD1, differing from mechanistic pathways in animal studies. From a clinical perspective, acral paresthesia and pain were reported 3 to 4 weeks after AAV gene therapy, accompanied by a reduction and then loss of sensory nerve action potentials. The post-mortem of a participant enrolled in the giant axonal neuropathy gene-therapy study, who did not demonstrate clinical symptoms or signs of DRG toxicity, identified severe neuronal loss within the DRG without inflammation. Sensory symptoms may be challenging to assess objectively, such that non-invasive and easily accessible translational biomarkers are attractive and possess the potential to improve monitoring and early diagnosis. Even so, coupling these with long-term longitudinal clinical data is needed to understand the relevance. Therefore, appropriate assessment and interpretation of symptoms, examination, and neurophysiology remain critical.
